# Statistical model of trajectories of reduced mobility in older people with locomotive disorders: a prospective cohort study with group-based trajectory model

**DOI:** 10.1186/s12877-023-04405-z

**Published:** 2023-10-30

**Authors:** Tsutomu Iwaya, Hideki Tanabe, Yusuke Ohkuma, Ayumi Ito, Kunihiko Hayashi, Yuki Ideno, Kazue Nagai, Masami Akai

**Affiliations:** 1 Nagano University of Health and Medicine, 11-1 Imaihara, Kawanakajima-chou, Nagano-shi, Nagano, 381-2227 Japan; 2 Tanabe Orthopaedic Clinic, 3-2-16 Narimasu, Itabashi-ku, Tokyo, 175-0094 Japan; 3 National Rehabilitation Center for Persons with Disabilities, 2-1 Namiki, Tokorozawa-shi, Saitama, 359-8555 Japan; 4 Takasaki University of Health and Welfare, 501 Nakaorui-machi, Takasaki-shi, Gunma, 370-0033 Japan; 5 School of Health Sciences, Gunma University, 3-39-22 Showa-machi, Maebashi-shi, Gunma, 371-8514 Japan; 6 Center for Mathematics and Data Science, Gunma University, 3-39-22 Showa-machi, Maebashi-shi, Gunma, 371-8514 Japan; 7 International University of Health and Welfare, Graduate School, International University of Health and Welfare, 4-1-26 Akasaka, Minato-ku, Tokyo, 107-8402 Japan

**Keywords:** Musculoskeletal disorders, Locomotive disorders, Geriatric locomotive function Scale-25, Group-based trajectory modeling, Functional outcome

## Abstract

**Background:**

Today we experience “Super-aged society”, and a drastic increase in the number of older people needing assistance is an urgent matter for everyone from medical and socio-economical standpoints.

Locomotive organ dysfunction due to musculoskeletal disorders is one of the main problems in these patients. Although the concepts of frailty and sarcopenia have been proposed for functional decline, pain remains the main and non-negligible complaint in these of such disorders.

This prospective cohort study aimed to observe the changes of reduced mobility in patients with locomotive disorders and to determine the risk factors for functional deterioration of those patients using statistical modeling.

**Methods:**

A cohort of older adults with locomotive disorders who were followed up every 6 months for up to 18 months was organized. Pain, physical findings related to the lower extremities, locomotive function in performing daily tasks, and Geriatric Locomotive Function Scale-25 (GLFS-25) score were collected to predict the progress of deterioration. Group-based trajectory analysis was used to identify subgroups of changes of GLFS-25 scores, and multinomial logistic regression analysis was performed to investigate potential predictors of the GLFS-25 trajectories.

**Results:**

Overall, 314 participants aged between 65 and 93 years were included. The participants were treated with various combinations of orthopedic conservative treatments on an outpatient basis.

The in-group trajectory model analysis revealed a clear differentiation between the four groups. The mild and severe groups generally maintained their GLFD-25 scores, while the moderate group included a fluctuating group and a no change group.　This study showed that comorbidity of osteoporosis was related to GLFS-25 score over 18 months. Age was a weak factor to be moderate or severe group, but gender was not. In addition, the number of pain locations, number of weak muscles, one-leg standing time, grip strength and BMI significantly contributed to the change in GLFS-25 score.

**Conclusions:**

This study proposes an effective statistical model to monitor locomotive functions and related findings. Pain and comorbid osteoporosis are significant factors that related to functional deterioration of activities. In addition, the study shows a patient group recovers from the progression and their possible contributing factors.

## Background

Japan has entered a “super-aged society”, with an aging rate of 29.1% of the entire population of the country. As of September 15, 2021, there were approximately 36.40 million people aged 65 years and over [1]. The number of people needing long-term care is rapidly increasing, and the number of people aged 65 years and over who are officially certified as requiring long-term care is 6.88 million (5.5% of the total population) as of September, 2021 [2].

This has a great influence on Japanese socio-economic situation. A decrease in the total population combined with an increase in the older population, which has a heavy financial burden, makes a significant impact on the society. Wallace proposed a term “age quake” by analogy with an earthquake to show its impact [3].

The Japanese Government has developed long-term care insurance as a health support system, combined with medical insurance, to respond to an aged society. Among various diseases that lead to conditions requiring long-term care, locomotive disorders have garnered increasing attention from the elucidation of pathophysiology with pain as the main complaint [4]. Unlike dementia, which is another condition that requires long-term care service, pain and locomotive disorders can be treated to a considerable extent due to their long history in the field of orthopedics.

To address this critical problem, Geriatric Locomotive Function Scale-25 (GLFS-25), a self-reported scale on activity of daily living (ADL) disabilities [5], was developed, and a survey was conducted to obtain the national standard values by age group and gender [6]. The deterioration pattern of ADL difficulties and physical impairments, detected using a self-filling questionnaire and motor function tests, have been reported to some extent [7–9].

Recently if longitudinal or time-series data at three or more time points are available, it is possible to statistically analyze the trajectory of changes that incorporate both individual and group differences [10]. Currently, early changes in locomotive disabilities in older people can be identified from longitudinal studies by means of statistical modeling.

This study aimed to observe the change of reduced mobility and to investigate the risk factors for functional deterioration in older people with locomotive disorders using group-based trajectory model (GBTM) [11, 12].

Early detection of individual’s inability to perform activities in everyday life is critical. Longitudinal studies of older people who need long-term care due to decreased motor function were conducted to identify the risk factors, motor function tests that could detect the initial changes, and how disease progression could be prevented or recovered.

## Methods

A cohort of five hospitals on an outpatient basis was organized. Data collected from a prospective study on the (1) disablement process of locomotive disability and (2) development of a prevention method for locomotive disability were used (“LDP study” supported by a Sciences Research Grant from the Ministry of Health, Labor and Welfare, Japan (H21 - Choju - G006)). We have already published a part of the above study [9, 13, 14], and analyzed the data collected every six months for up to 2 years in the current study.

### Recruitment of participants

Patients aged ≥ 65 years were recruited from five orthopedic clinics and affiliated nursing care facilities. Written informed consent was obtained from all participants.

#### Inclusion criteria

1. Age ≥65 years (either gender)
2. Any one of the following 4 criteria
  - Complaints related to the lower extremities or spine with no disability in walking or leaving the home (outpatients).
  - Complaints related to the lower extremities or spine, with slight disabilities in walking or leaving the home (outpatients).
  - Slight disability in walking due to locomotive organ disorders (users of long-term care services).
  - Complaints related to the upper extremities without disability in walking or leaving the home (outpatients at orthopedic clinics).
3. Ability to answer the GLFS- 25 questionnaire without assistance
4. Consent to radiographic examination of the knees and spine
5. Consent to examination of serum vitamin D and hyaluronic acid levels
6. Consent to participate in the following motor function tests: one-leg standing, grip strength, leg extension power, 50-steps test, and trunk forward bending test

#### Exclusion criteria

1. Unable to stand up from chair or bed without help.
2. Walking or locomotive disability because of brain disease requiring treatment at the time of admission.
3. Severe pulmonary, renal, coronary, or hepatic diseases.
4. Mental illness.
5. Past history of stroke within the preceding 6 months.
6. Past history of myocardial infarction within the preceding 6 months.
7. Past history of fracture of the lower extremity within the preceding 6 months.
8. Current treatment for acute trauma.
9. Other reasons determined by the attending physician.

### Data collection

The participants were asked regarding previous history of falls (past one year) and fractures, regular medications, diagnoses related to the locomotive organs, comorbidities, use of walking aids, living environment (especially number of family members), and orthopaedic interventions, and to complete the GLFS-25 questionnaire.

Attending physicians examined the patient’s complaints and the area of pain (back, buttock, thigh, or knee), neurological signs, determined the posture classification, and recorded the physical findings related to the trunk and lower extremities. The staffs also measured the body height, body weight, range of motion (ROM) of the hip and knee joints, and strength of the iliopsoas, quadriceps, anterior tibialis, and calf muscles and administered and recorded the results of the motor functional tests, including one-leg standing time, grip strength, leg extension power, 50-steps time, and trunk forward bending distance.

The staff obtained the radiographs, including the anteroposterior view of the knee joints in a standing posture and the lateral view of the thoracolumbar spine, and assessed them quantitatively using a semi-automated computer-aided diagnosis [15]. The bone density of the wrist or metacarpal bones, lumbar spine, or proximal femur was measured, and expressed as a percentage of the mean in young adults. Serum vitamin D and hyaluronic acid levels were also measured.

The patients participated in outpatient rehabilitation programs at the aforementioned five facilities and were examined four times: at baseline and after 6, 12, and 18 months. In this study, the baseline data were predominantly used except for GLFS-25 scores.

### Creation of variables

The present study aimed to operationally define locomotive disorders (knee osteoarthritis, lumbar spondylosis, and osteoporosis) using parameters such as pain (low back, gluteal, posterior thigh region, and knee joint), physical findings related to the lower extremities (ROM, muscle strength, sensory impairment, and deep tendon reflex), locomotive function (balance, lower limb muscle power, trunk flexibility, and ability to step in place), and GLFS-25 score.

#### Muscle strength

Physiotherapists evaluated the muscle strength of the bilateral iliopsoas, quadriceps, anterior tibialis, and calf muscles using manual muscle testing (six grades).

If a muscle scored 5 on both sides, its strength was classified as normal; otherwise, it was classified as weak.

#### Pain sites

Attending physicians examined the lower back, buttocks, bilateral posterior thigh region, and knee joints regardless of whether an individual reported soreness, tenderness, or pain during motion in these areas.

If the patient did not complain of lower back pain (LBP), LBP was classified as “-”, otherwise as “+.” For pain in the buttocks, posterior thigh, or knee joints, if the patient did not complain of pain on either side, pain in the relevant region was classified as “-” otherwise as “+.” We counted the number of pain locations (1–6).

#### Physical functions; motor function tests

The one-leg standing test measures the time (sec) which the subject can stand on one-leg with the eyes open. One-leg standing time was measured bilaterally and the mean of the two trials was calculated.

Grip strength was measured bilaterally using a dynamometer (kg) and the stronger value of the bilateral hands designated grip strength.

Leg extension power was measured bilaterally using a device to measure the extension force (kg) against force plate vertically set. Stronger value from the two trials was designated as leg extension power.

The 50-steps test measures the time (sec) required to step in place 50 times. The shorter value from the two trials was designated as 50-steps time. This was used to enable observations even in small examination rooms.

The trunk-bending test measures the distance (cm) between the fingertip and foot sole as the subject bends forward as deeply as possible in a long sitting posture. The longer distance between the two trials was designated as the trunk-bending distance.

The 10 fields, 42 items, and 392 variables assessed in this cohort study are shown in Table 1.

**Table 1:** List of variables used for data creation and analysis

Areas	Item numbers		Variable numbers
Basic information	**8**	**Gender/Age**, occupation, education, certification for long-term care, certification for specific elderly, Body measurements (height/weight, **BMI**)	7
living environment	**2**	cohabiting family, residence	8
health condition	**5**	Cognitive, emotional, vision, hearing, use of walking aids	25
Medical history: Comorbidities	**3**	Medical history, comorbidities, regular medications	42
Medical history: Locomotive disease	**4**	Chief complaint, musculoskeletal disease, details of treatment, fracture history	65
Physical findings for back/lower limbs	**4**	**Pain by site** (back pain, buttock pain, thigh pain, knee pain) Posture, knee, neurological findings, functional classification, ROM, MMT	19
laboratory Examination	**5**	Vitamin D, Hyaluronic Acid, Bone Mineral Density	3
XP measurement	**2**	X-P (knee joint, lumbar spine)	159
motor function tests	**8**	**Grip strength**, long sitting forward bending distance, 50 step stepping time, **(eye open) one leg standing time**, leg extension force	39
**GLFS-25**	**1**	**25 questions**	25

Three hundred and ninety-two variables are extracted from collected data. Variables used for GBTA and Multinomial logistic regression analysis are shown in bold

*BMI *Body mass index, *ROM *Range of motion, *MMT *Muscle manual test, *GBTA *Group-based trajectory analysis

### Principal scale for assessment: geriatric locomotive function Scale-25

The GLFS-25 is a self-administered measure consisting of 25 items. These items are graded on a 5-point scale from no impairment (0 points: not difficult to do) to severe impairment (4 points: difficult to do), and then arithmetically added to produce a total score (minimum 0 and maximum 100).

The participants were asked to complete the GLFS-25 questionnaire. As previously mentioned, the reliability and validity of the GLFS-25 have been certified [5] and the national standard value of the GLFS-25 has also been reported [6],

The questionnaire consists of 25 items asking about status over the last month, including 4 questions regarding pain, 3 regarding mobility, 5 regarding self-care, 4 regarding locomotion, 3 regarding housework, 4 regarding joining social activities, and 2 regarding mental health.

Despite its screening role in the development process, the GLFS-25 has proven to clearly reflect the severity of support or care-need levels [13]. Using this scale, we attempted to determine the characteristics of impairment of functional activities and limitation of social participation, or, if possible, their consistent common patterns of deteriorations.

### Analytic methods

In recent years, if longitudinal or time-series data at three or more time points are available, it is possible to statistically analyze the trajectory of changes that incorporate both individual and group differences within them. There are many methods with various names, such as the latent growth mixed model, latent growth curve model, or hierarchical linear model; however, they are included in the method of structural equation modeling [10].

Conventional longitudinal trajectories were considered to account for individual variability in the mean population trend. However, one of such statistical methods, the Group-based trajectory models (GBTM) is a “statistical portrait” of the predictors and consequences of distinct subgroup trajectories of development or progression [11, 12, 16]. Jones and Nagin released the GBTM computer program adapted to the STATA platform SAS-based procedure [17].

The GBTM is a specialized application of finite-mixture models, which is a statistical model that assumes the presence of unobserved groups, called latent classes, within an overall population. Such a trajectory analysis is a method of finding latent classes based solely on changes in the GLSF25 score, rather than comparing things.

In addition, from variables such as the number of pain sites, the number of muscles with weakness, comorbidities, 5 motor function tests, body mass index (BMI) and age, we selected explanatory variables for which calculation convergence was obtained using statistical software. While the conceptual aim of the analysis is to identify clusters of individuals with similar trajectories, the estimated parameters of the model are not the result of cluster analysis.

First, using GBTM, GLFS-25 scores were analyzed among participants who provided the GLFS-25 scores four times to identify subgroups of GLFS-25 score changes. The Akaike Information Criteria (AIC) was used to determine the best model of trajectory subgroups. A sensitivity analysis was performed using multiple imputation trajectory model to account for missing data in participants who provided GLFS-25 scores at least twice.

Second, multinomial- logistic regression analysis was performed to explore factors that determine changes in longitudinal data among such groups, using the GLFS-25 score as the dependent variable, and the age, gender, presence/absence of osteoporosis, number of painful areas, number of muscle weakness, BMI, number of seconds to stand on one-leg with eyes open, and grip strength were used as independent variables. Except for GLFS- 25 scores, the baseline data were also used in this regression analysis.

### Statistical software

Data analyses were performed using SAS software, version 9.4. software (SAS Institute, Cary, NC, USA). The SAS macro PROC TRAJ was used to analyze the GLFS-25 trajectory to indicate longitudinal changes in the GBTM [18].

## Results

### Demographic data of the participants at baseline

Overall, 314 participants (80 men and 234 women) aged between 65 and 93 years were included. The mean age was 75.9 years (standard deviation [SD] = 6.3) in men and 77.9 years (SD = 8.0) in women. The mean of the GLFS-25 scores was 23.0 (range, 0–73; SD = 15.8).

The diagnoses of the participants were knee osteoarthritis (*n* = 136), osteoporosis (*n* = 67), spinal canal stenosis (*n* = 58), spinal spondylitis (*n* = 54), and multiple diagnoses (*n* = 133). Of the 314 participants, 268 had comorbidities, such as hypertension or diabetes.

In addition, 143 participants used walking aids, such as sticks, canes or walkers. A history of falling was reported by 233 participants, and 158 had history of fractures within the past few years. More detailed data other than musculoskeletal conditions on the basic characteristics of the participants have been previously reported [13, 14].

### Participant follow-up

During the18-month follow-up, 89 patients dropped out; remaining 225 patients, (59 men and 166 women) were completed the study and successfully provided 4 times data. However, there were missing data even in these completed cases, with 220 cases of four-time complete data on the central GLFS-25 score, and 217 to 225 cases on motor function tests, among others.

### Data cleaning of the collected dataset

In the four GLFS-25 longitudinal evaluations, there were missing values in 9 cases and 12 items that were corrected using the carry over method.

As for missing data of 50-steps time, grip strength, forward trunk-bending, one-leg standing with eyes open, and BMI, the average was calculated using before and after values.

### Contents of orthopaedic interventions

**Fig. 1:**
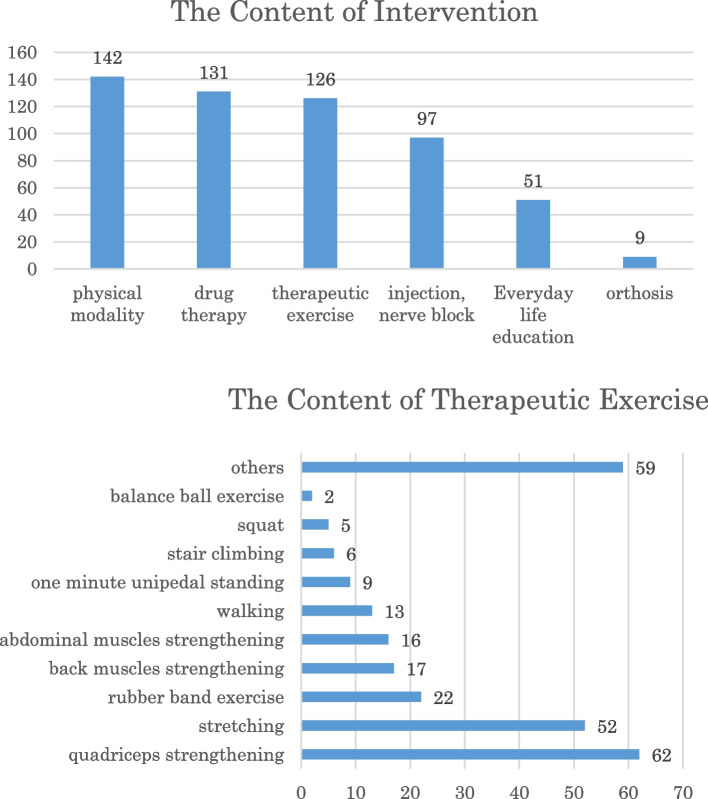
The Content of orthopaedic interventions for the participants. The figure shows the treatment content of the participants who were undergoing orthopedic outpatient care. The treatment content was not controlled for this study in the protocol

Figure 1 shows the orthopaedic interventions for the participants. Regarding drug therapy, non-steroidal anti-inflammatory drug were mainly used for pain control. And therapeutic exercise (there is overlap) shows the actual situation.

These were no regulations on their assignment in the current study.

### Scores at the baseline among the GLFS-25 levels

**Fig. 2:**
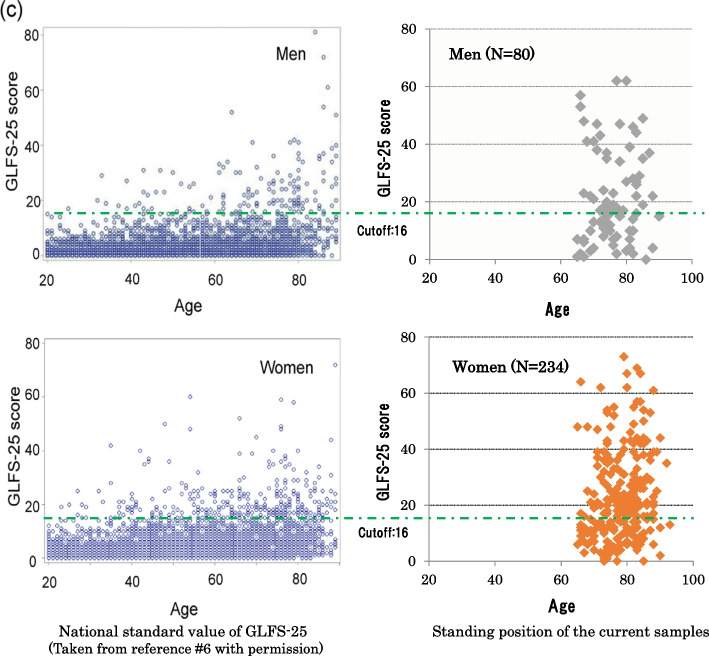
Standing position of the current samples in the national standard values of GLFS-25. The figure shows the distribution of participants in this study according to the GLFS-25 national norm by gender. The cutoff score for identifying clinical manifestation of locomotive disorder was set at 16 (taken from reference [5])

Figure 2 shows the standing position of the current samples in the national standard distribution. As the national standard value of GLFS-25 has been investigated, it shows the level of each case at the start of this survey compared to the Japanese standard value.

It is evident that our samples were included slightly more decreased group, and was distributed in the age groups at which clear symptoms began.

### Trajectories of reduced mobility and In-group tendency

The final 220 participants that provided the GLFS-25 scores four times were used in the calculation for GBTM out of the baseline 314 samples.

The in-group trajectory model analysis revealed a clear differentiation between the four groups: mild group (36.3%), moderate group with no change (13.1%) or with deterioration and recovery (12.5%), and severe group (38.1%). The sensitivity analysis, in which missing GLFS-25 scores were imputed (*n* = 289), and confirmed that the results were almost the same in imputed cases (data not shown). The AIC showed a better fit for the 4-group model (AIC = -3112.33) rather than the 3-group one (AIC = -3106.57).

**Fig. 3:**
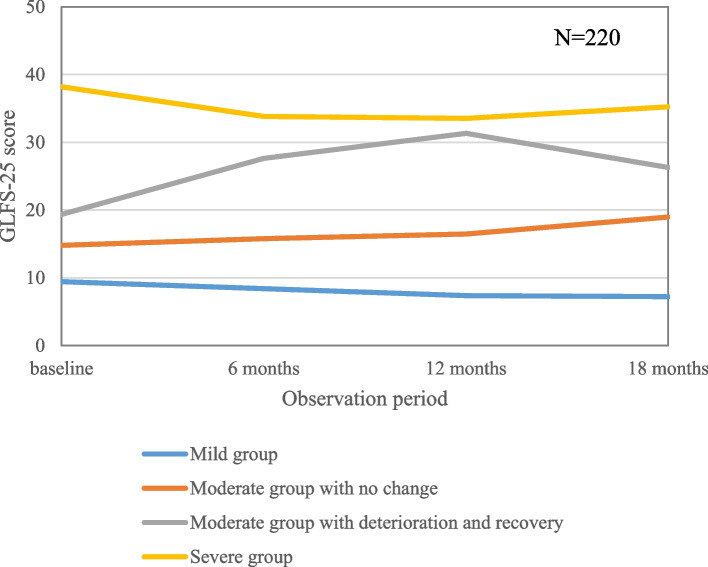
Trajectories of the four groups to the GLFS-25 score changes. The trajectory analysis was divided into four groups. While many of the mild and severe groups remained unchanged, the moderate group was roughly divided into two groups: those that remained unchanged and those that showed changes in exacerbation/improvement

The mild and severe groups generally showed maintained GLFS-25 scores, however, the moderate group included a fluctuating group that showed deterioration and improvement. The two moderate groups had the same starting point, but different outcomes (Fig. 3).

Table 2 shows the results of the multinomial logistic regression analysis. For each GLFS-25 subgroups variable, the mild group was used as the reference group.

**Table 2:** Multivariable analysis of baseline factors associated with GLFS-25 trajectory

	Moderate group with no change vs. Mild group		Moderate group with deterioration and recovery vs. Mild group		Severe group vs. Mild group	
**Variables**	OR	(95% CI)	OR	(95% CI)	OR	(95% CI)
Age	1.043	(0.957, 1.137)	**1.148**	**(1.042, 1.265)**	**1.080**	**(1.005, 1.161)**
Gender:						
F	1	(reference)	1	(reference)	1	(reference)
M	1.535	(0.331, 7.116)	1.728	(0.298, 10.014)	2.219	(0.622, 7.915)
Osteoporosis:						
No	1	(reference)	1	(reference)	1	(reference)
Yes	**3.920**	**(1.250, 12.300)**	0.463	(0.080, 2.685)	2.848	(0.986, 8.223)
Number of pain locations	**1.429**	**(1.024, 1.992)**	1.427	(0.999, 2.038)	**1.820**	**(1.363, 2.430)**
Number of weak muscles	0.831	(0.631, 1.095)	**0.666**	**(0.480, 0.924)**	1.060	(0.836, 1.345)
BMI	0.980	(0.836, 1.149)	1.164	(0.989, 1.370)	**1.158**	**(1.016, 1.321)**
One leg standing time	0.975	(0.951, 1.000)	**0.966**	**(0.935, 0.997)**	**0.961**	**(0.937, 0.985)**
Grip strength	0.945	(0.852, 1.048)	**0.858**	**(0.756, 0.973)**	0.934	(0.854, 1.023)

The GLFS-25 trajectory was categorized into 4groups, and the factors at the first time (reference group 1) from the multinomial logistic analysis are shown

Multinomial logistic regression analysis was four groups of GLFS-25 as dependent variables; several baseline (the first time) data (age, gender, presence or absence of osteoporosis, number of painful areas, number of weak muscles, BMI, one leg standing with eyes open, grip strength) as independent variables

Number of pain sites (0 ~ 7): low back pain, bilateral gluteal pain, bilateral posterior thigh pain, bilateral knee pain. Number of muscles with weakness (0 ~ 8): bilateral iliopsoas, bilateral quadriceps femoris, bilateral tibialis anterior, bilateral triceps surae

Bold indicates a significant odds ratio which 95% CI does not distribute around 1

*OR *Odds ratio

*CI *Confidence interval

*BMI *Body mass index

Age was a weak factor to be moderate or severe group, but gender was not. In addition, the number of weak muscles, one-leg standing time, grp strength, and BMI significantly contributed to the change in GLFS-25 score.

Compared to the mild group, the moderate group with no change group had 3.92 odds of presence of osteoporosis and 1.43 odds of number of painful areas.

On the contrary, the moderate group with deterioration & recovery group had 1.15 odds of baseline age, 0.67 odds of number of painful areas, 0.97 odds of one-leg standing time, and 0.86 odds of grip strength. This group had 0.46 odds of presence of osteoporosis.

Compared to the mild group, the severe group had 1.08 odds of baseline age, 1.82 odds of number of painful areas, 1.16 odds of BMI, and 0.96 odds of one-leg standing time.

If there is comorbidity of osteoporosis, it is more likely to be in the moderate group with no change group compared to the mild group. The association with the severe group was not statistically significant; however, the trend was different from that of the deterioration and recovery groups (OR, 2.85 and OR, 0.46 respectively).

## Discussion

It is necessary to develop intervention strategies that could delay the decline of functional ability in healthy people, as well as to recover the functioning state in people who have been disabled due to aging. In addition, long-term follow-up of patients in this age group is not easy because of the increased death rate [19]. Therefore, an appropriate mathematical statistical model is required.

The idea of explaining aging through the accumulation of various age-related changes has been previously reported and especially in the last year of life, several distinct trajectories were identified. Gill et al. identified disabling process follows a general pattern of progression based on a typical sequence of impairments, but the course of disability in the last year of life did not follow a predictable pattern [20]. On the other hand, those who died at the oldest ages were much more likely to have disability 2 years before death [21]. Understanding such a pattern of functional deterioration, it has helped illuminate mechanisms of impairment and inform interventions, but little is known about locomotive disorders by age.

Hence, it is important to determine the risk factors for functional deterioration among older adults to prevent them from falling into long-term care. Elucidation of the early, initial changes and identification of risk factors using statistical models will be extremely meaningful.

The results of the current study proved that complaints of pain are significant in musculoskeletal disorders. Both the severity of pain and the extent of any accompanying disability are key factors in assessing musculoskeletal disorders [22]. Careful attention should be paid to pain management in these patients.

To develop intervention strategies for older adults with disability and those who need assistance with ADLs, it is mandatory to investigate the impairments, functional abilities and disabilities in people with locomotive disorders, and to examine the relationships between the parameters.

Locomotor disorders have gained increasing attention, as a result, an intervention strategy to improve prognosis in people with disability caused by locomotor dysfunction and for those who need nursing care tis highly expected.

### Purpose of medical examination for prevention

The World Health Organization has released the guideline for medical examination [23]. Wilson and Jungner proposed 10 principles of early disease detection (case-finding) and we referred five important principles.

Target diseases to be examined must;

1. have “significance to the individual and the community”;
2. have “recognizable latent or early symptomatic stage”;
3. have “facilities for diagnosis and treatment”;
4. have “suitable test or examination”; and
5. ensure that the natural history of the condition, including development from latent to declared disease, should be adequately understood.

The purpose of a medical examination is not to obtain a diagnosis, but to treat it and change its prognosis. Diseases that are cured in a short period are not included by the target of medical examinations. Moreover, there should be feasible primary screening and secondary precise examination available for identifying abnormal cases. Certain treatment options are required. Unless we can prevent or delay the disease, we cannot assume that we have changed the prognosis.

Diagnostic techniques and treatments, including various surgeries, for locomotor disorders or orthopaedic disease have long been established. When assessing locomotor disorders and patient meet the above requirements, we have the ability to change the prognosis. Unlike intellectual impairment in older people, in which it is hard to recover fully, the methods we can use have some effectiveness in the treatment of locomotor disorders.

The effectiveness of general health check appears to be skeptical, and controversies over their efficacy continue [24–27]. We must give due consideration to the nature of the disease in question and narrow down the application target.

Crimmins advocates the morbidity process model [28], in which even physiological changes (such as blood sugar and cholesterol levels) lead to illness and changes in physical condition, thus resulting in frailty, functional loss, disability, and even death. This broadens the horizons of the disablement model.

### Pain in locomotive disorders

The prevalence of musculoskeletal pain is high among the elderly population. According to the 2019 National Livelihood Survey, the prevalence of low back pain among the elderly people aged 65 and over per 1,000 populations was 91.2 for men and 113.8 for women, and 41.3 for men and 62.9 for women with limb joints pain [29]. In addition, joint disease is ranked first as a causative disease requiring support in the long-term care system in Japan [4].

Musculoskeletal pain influences the activities of the responsible organ. An increase in the number of pain sites increases the number of restricted activities, leading to a decline in physical functioning [30]. The results of this study, which clarified the relationship between transitions in the GLFS-25 scores, that is, transitions in activity restrictions and the number of pain sites, indicate the significance of pain treatment as a medical intervention to prevent nursing care and worsened nursing care conditions.

Similar or wider clinical concept such as “frail” covers multiple organ deteriorations due to aging.

Although there are several definitions of frailty and sarcopenia, the concepts of decreased muscle mass and strength, and decreased function in multiple organs have similar characteristics [31, 32]. No factor was associated with functional deterioration due to pain.　However, the role of pain in public health has been emphasized in recent years [22] as well as in this study.

We found that not only chronic pain significantly impairs ADL, pain-centered musculoskeletal problems could be also associated with functional decline from the current longitudinal data over time. We have reported that the GLFS-25 score was related to the number of locomotive impairments (knee pain, low back pain, ROM limitation and muscle weakness of the lower extremities) and motor functional ability (grip strength, one-leg standing and leg extension power) [33].

### Selection of the explanatory variables

When performing calculations to obtain a statistical model, it is important to consider how to incorporate appropriate explanatory variables. Recently, several datasets have been proposed for screening or assessment tool for older people. A proposal for frailty index [34] or a simple tool for screening of sarcopenia called SARC-F [35, 36] was noticed and its contents were checked.

Some researchers have described these as core datasets or core outcome measures [37, 38]. This method should be simplified to consider its feasibility. We followed these trends and selected the items. A consensus of the necessary dataset had once been established, and we could compare future studies.

### Healthy life expectancy

The 2022 survey results of the Ministry of Health, Labor, and Welfare reported that the average life expectancy of Japanese men and women is 81.47 and 87.57 years respectively, and a healthy life expectancy is 72.68 and 75.38 years respectively [39].

The difference is 8.79 years for the men and 12.19 years for the women. The report suggests that both men and women have been living their lives with physical disability or have been bedridden for approximately 10 years.

Considering such changes beginning after 70years of age, it is important not only longer follow-ups, but to adopt appropriate statistical prediction models to determine how healthy life expectancy would be lost. In this age group, long-term follow-up may be difficult.

### Classification of targeted older adults

Recently there has been a movement towards common-data elements and core outcome measures in frail research [36]. We believe that such movement could be of great help for locomotive diseases.

Progressive disability develops with older age and is often associated with underlying diseases, comorbidities, and other complicated conditions. Hence, it is important to classify older people in order to predict future progression of motor dysfunction [32].

Considering the results of the current study, there was no change in trend in three of the four groups, but a recovery trend was observed in one of the intermediate groups. The ability to change the prognosis is an important condition for disease prevention, and we believe that this possibility has been demonstrated.

Iwaya et al. reported a certain deterioration pattern observed in each item of the GLFS-25 [14], however, it was a pattern derived from the score distribution, not from the actual longitudinal data. The current study revealed the changes over time and determined the items that have the potential for recovery. Although it cannot be strictly defined as the natural course without treatment, it is important to be able to capture the course that many elderly people take.

### Orthopaedic intervention

The majority of the participants in this study were treated with various interventions including drugs, physical modalities, and therapeutic exercise. The findings suggest that a certain intervention improves locomotive function to some extent even in out-patients base, however, we should also note differences in responses among subgroups of locomotor disorders.

However, the current analysis is a cohort observational study and there was no controlled intentional treatment.

## Limitation of the present study

This prospective cohort study was conducted for up to 2 years. Participants generally received various orthopaedic conservative treatments; however, their indications were not regulated in this study.

A longer-term follow-up of more than 2 years should ideally confirm that the orthopaedic interventions improve the prognosis of the target patient and that the preventive interventions triggered by medical examinations are meaningful.　If the risks are properly understood, the intervention methods we currently have, such as exercise, drugs, and even surgery, may be effective for patients like those in this study.

## Conclusions

We proposed an effective statistical model to monitor locomotive functions and the related findings. Pain and comorbid osteoporosis are significant factors that contribute to functional deterioration of activities. We also found a patient group recovers from the progression and their possible contributing factors.

If we make good use of our treatment experience in the orthopedic field for locomotor disorders, including analgesic drugs, exercise or even surgery, it could be possible to prevent the progression.

## Data Availability

The datasets generated and/or analyzed during the current study are available from the corresponding author upon reasonable request.

## Abbreviations

ADL: Activity of daily living
GBTM: Group-based trajectory modeling
GLFS -25: Geriatric Locomotive Function Scale – 25
LTCI: Long-term care insurance
ROM: Range of motion
MMT: Muscle manual test
% YAM: % young adults mean
AIC: Akaike Information Criteria

## Declarations

Written informed consent was obtained from all participants, and all study protocols were approved by the Institutional Review Board of the National Rehabilitation Center for Persons with Disabilities (No. 21–77).
